# Global Health Implications of US Exit From WHO and Suspension of Aid

**DOI:** 10.1002/puh2.70267

**Published:** 2026-05-05

**Authors:** Kenneth Chukwuebuka Egwu, Maryam Abdulkarim, Molly Unoh Ogbodum, Yusuff Adebayo Adebisi, Dukanwojo Beulah Suleman, Don Eliseo Lucero‐Prisno

**Affiliations:** ^1^ Department of Pharmacy Karshi General Hospital Karshi, Abuja Nigeria; ^2^ The Community Bridge Health Initiative, Awka Anambra State Nigeria; ^3^ Department of Clinical Infection Microbiology and Immunology University of Liverpool Liverpool UK; ^4^ Department of Public Health University of Calabar, Calabar Cross River Nigeria; ^5^ College of Social Sciences University of Glasgow Glasgow UK; ^6^ Department of Sociology University of Nebraska‐Lincoln Lincoln Nebraska USA; ^7^ Faculty of Public Health and Policy London School of Hygiene and Tropical Medicine London UK; ^8^ Faculty of Management in Public Health and Pharmacy Asfendiyarov Kazakh National Medical University Almaty Kazakhstan; ^9^ Faculty of Management and Development Studies University of the Philippines Open University Los Banos Laguna the Philippines

Upon assuming office, President Donald Trump, on January 21, 2025, signed an executive order to withdraw the United States (US) from the World Health Organization (WHO) [1]. The unprecedented decision undermines multilateralism at a time when global challenges, such as cross‐border and planetary health threats, are becoming more pressing. In 2020, Trump expressed his intention of withdrawing the US from the multilateral organization due to concerns about mishandling COVID‐19 response [1]; however, his current administration has reinitiated the process with an unyielding commitment. This action has profound implications for the global health community and is further complicated by the government's halting of international aid across various health programs. This decision has prompted deep reflection among global health leaders, governments, and citizens—particularly in low‐ and middle‐income countries (LMICs)—as they risk losing years of progress in combating diseases such as HIV/AIDS, tuberculosis, malaria, polio, and other neglected tropical diseases. Although the aid suspension appears most visible, the US’ commitment to global health has been fragile for years. In 2023, Congress failed to renew the US President's Emergency Plan for AIDS Relief (PEPFAR) over abortion‐related politics, whereas the United States Agency for International Development (USAID) had already shifted from broad health priorities toward security‐driven agendas, markedly increasing pandemic preparedness funding while maternal and child health investments stagnated [2].

In 2004, the United Nations and the WHO warned that the futures of millions of children in six African countries were being jeopardized by severe funding shortfalls and insufficient international support for critical aid projects [3]. This underscores the critical importance of ensuring adequate and sustained resourcing for multilateral agencies. The importance of the US to WHO and global health cannot be overemphasized. Although WHO obtains its funding through two major routes, member states’ mandatory fees (assessed contributions) and voluntary contributions from member states and partners [4], the US provides a bulk of this funding. In 2023, it remitted 18% of the total WHO funding and 22% of the total $6.8 billion WHO budget for 2024–2025 fiscal year [1], similar to its support in the 2022–2023 fiscal year (Figure 1) [5].

**FIGURE 1:**
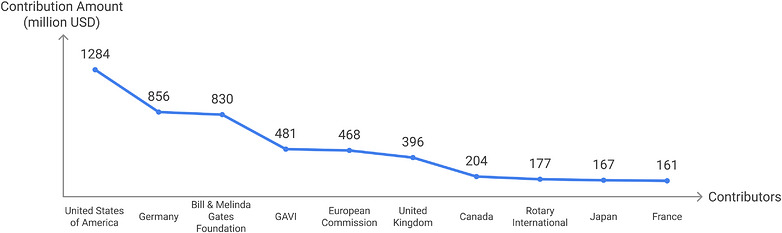
Top 10 contributors to WHO (2022–2023).

For decades, the country has shouldered the weight of global health leadership, strengthening health security, especially in countries with insufficient domestic funding. The US exit may impact global health diplomacy, including the potential leadership shifts with countries like China or the European Union stepping up [6].

The WHO has utilized funding and expertise of numerous US agencies and departments, including the US Department of Health and Human Services, the Centre for Disease Control, the Environmental Protection Agency, PEPFAR, and USAID to improve operations and pandemic response to successfully combat major health crises, such as HIV, tuberculosis, malaria, neglected tropical diseases, global health security threats, and maternal and child health challenges among others [7]. Notable is the $120 billion invested in PEPFAR by the government since its inception in 2003—making it the largest commitment by any country to eradicate HIV [8].

Withdrawal of the US from WHO and the suspension of global health aid could compromise global health initiatives such as the Global Fund and Global Alliance for Vaccines and Immunization (GAVI) which are heavily reliant on the US funding. The impact of these organizations is most evident in poor countries, where the Global Fund has saved an estimated 65 million lives and GAVI is facilitating the vaccination of more than 1.1 billion children in 78 LMICs and averting more than 18.8 million future deaths [9]. Through PEPFAR, the US government provides the largest donations to Global Fund, contributing $27.61 billion to date [10]. Alarmingly, if the country halts the provision of free anti‐retrovirals, made possible through PEPFAR, HIV/AIDS response may become limited unless countries rise up to address the challenge. A modeling study indicates that a complete withdrawal of US funding, without replacement from other sources, would result in substantial increases in mortality between 2025 and 2030, including 4.1 million additional AIDS‐related deaths, 606,900 tuberculosis deaths, and 2.5 million excess child deaths across numerous countries, with Mozambique, Nigeria, South Africa, Tanzania, Uganda, and Zambia accounting for 2.3 million of the additional AIDS‐related deaths [11]. Research estimates that nine sub‐Saharan African countries, namely, South Africa, Zimbabwe, Malawi, Uganda, Kenya, Nigeria, Zambia, Tanzania, and Ethiopia, will need $261 billion to combat HIV between 2015 and 2050 [12]. This sets a significant challenge for the region where domestic funding for HIV is either nonexistent or insufficient. The African region is undoubtedly disproportionately affected, with 25.6 million people living with HIV [13]. Out‐of‐pocket expenditures as an alternative means of support would be inadequate as people living with HIV still struggle with socioeconomic factors such as stigma, poverty, and abuse.

Furthermore, malaria eradication efforts across the globe could be jeopardized. The US remains the highest malaria fund donor government, with $1.1 billion invested for malaria control and research purposes in 2023 [14]. These efforts resulted in the distribution of millions of mosquito nets and medications to various households. As inflation impacts the global economy, a reduction in mosquito net usage is anticipated if funding is not sustained. Tuberculosis incidence would worsen, as the US provides substantial funding for its control through the Global Funding [9, 11]. Vaccination efforts could stall, leading to resurgence of previously eradicated diseases such as polio and measles. Even worse, this could result in a global economic crisis, heightening cross‐border disease spread and mortality.

Although the US decision to withdraw from WHO and suspend international health aid poses a significant threat to global health security, it underscores the need for countries and multilateral organizations to rethink funding strategies and prioritize sustainable health investments—a bold step that has been observed with the recent expansion of Brazil, Russia, India, China, and South Africa (BRICS) to other nations, including those in Africa, South America, and Asia. This broadened membership base offers an opportunity to democratize global health financing by reducing overreliance on traditional power blocs and mitigating the risks posed by unilateral decision‐making. Through instruments like the BRICS Development Bank and the BRICS Vaccine Research and Development Centre, the bloc can play a transformative role in supporting public health initiatives and expanding access to essential medicines, reinforcing collective self‐reliance in health security. Additionally, LMICs, many of which allocate more resources to debt servicing than to health expenditure, can leverage debt‐to‐health swaps (D2H) as an innovative financing mechanism to increase investment in the health sector. This approach would not only alleviate debt burdens but also make a significant contribution to disease prevention and the strengthening of global health systems [15]. Furthermore, to address the funding gaps posed by the global aid cut, African countries encompassing Nigeria, Ghana, and Kenya must reassess budget allocations, including reducing excessive expenses for political leaders and redirecting funds toward health initiatives. More so, governments should focus on driving local pharmaceutical manufacturing by reducing import duties on essential pharmaceutical ingredients and providing incentives for private‐sector investment. This will greatly ensure the availability of affordable vaccines, medications, and medical equipment and also bolster regional health resilience. Countries in the global south can also improve domestic resource mobilization and sovereignty by optimizing public spending, diversifying income sources, providing an enabling environment for private investors, promoting health innovation and entrepreneurship among citizens, and institutionalizing favorable taxes to attract and retain investors, especially in the health sector. In this regard, regional blocs such as the Association of Southeast Asian Nations (ASEAN) [16] and some South American countries [17] are increasingly taking ownership of health financing and preparedness and reducing reliance on external donors. Furthermore, public–private partnerships can play a crucial role by pooling resources from both sectors to advance health programs and research. Increased domestic investment in healthcare, including establishing dedicated health security funds, is essential to reduce dependency on external donors. Regional health alliances, such as the Africa Centre for Disease Control and Prevention, should be strengthened to facilitate and enhance homegrown coordinated responses to disease outbreaks and capacity‐building initiatives. The recent efforts to establish vaccine production hubs, such as the mRNA technology transfer program led by WHO in South Africa [18], represent promising steps toward self‐sufficiency and sustainable health security in Africa.

In light of these compounded threats, countries must prioritize investments to support health system resilience as a core component of national health security in response to the looming threats of the global aid cut. Strengthening community health systems through the deployment and fair compensation of community health workers and integrated mobile outreach and task‐shifting models has been shown to improve outbreak response and routine care delivery, particularly in hard‐to‐reach settings [19]. In addition, the threat of antimicrobial resistance (AMR) continues to grow in LMICs, fueled by weak surveillance, unregulated antimicrobial use, and fragmented funding. Addressing the growing AMR crisis hinges on sustained global research and development partnerships and stewardship networks paired with domestic commitments to antimicrobial stewardship and diagnostics. Without coordinated funding, AMR could cost an additional $1 trillion in health expenditures by 2050 [20]. Similarly, declining immunization rates exacerbated by vaccine hesitancy and aid withdrawal present the risk of resurgence for vaccine‐preventable diseases. Targeted risk communication and reinvestment in routine immunization programs are urgently needed to prevent reversal of past gains [21]. Building strong health systems in such a time as this will require all hands on deck to integrate preventive strategies into health governance frameworks.

The global ramifications of the US exit go beyond financial contributions. It threatens to undermine pandemic preparedness and response capabilities at a time when emerging infectious diseases are on the rise. Of particular concern is the weakening of global early warning and rapid response systems, essential for detecting and containing cross‐border outbreaks. The ongoing spread of H5N1 avian influenza in US dairy cattle—with signs of mammalian adaptation—illustrates the looming risk of zoonotic spillover and potential pandemic emergence across the one health framework [22]. To safeguard global health security, countries must adopt innovative, domestic, and collaborative approaches and build solidarity to shape a resilient and self‐reliant global health ecosystem. Failure to adapt could threaten global health gains and reverse years of progress in disease eradication and health equity, leaving the world ill‐prepared for future health crises.

## Author Contributions

**Kenneth Chukwuebuka Egwu**: conceptualization, writing – original draft, writing – review and editing. **Molly Unoh Ogbodum**: writing – original draft, writing – review and editing. **Yusuff Adebayo Adebisi**: writing – review and editing, supervision. **Dukanwojo Beulah Suleman**: writing – review and editing, supervision. **Maryam Abdulkarim**: writing – original draft, writing – review and editing, visualization. **Don Eliseo Lucero‐Prisno III**: writing – review and editing.

## Funding

The authors have nothing to report.

## Ethics Statement

The authors have nothing to report.

## Conflicts of Interest

Yusuff Adebayo Adebisi is an editorial board member of Public Health Challenges and co‐author of this article. To minimize bias, he was excluded from all editorial decision‐making related to the acceptance of this article for publication.

## Data Availability

Data sharing not applicable to this article as no datasets were generated or analyzed during the current study.
